# A First-Principles Study on the Adsorption and Desorption of Nitrogen on UN and U(N,C) Surfaces

**DOI:** 10.3390/ma19204304

**Published:** 2026-10-13

**Authors:** Panpan Duan, Wanying Li, Xiuli Han, Xiaoqiang Pan, Jing Zhang, Yonghong Lu, Danke Su, Rui Wang, Yujin Wang, Lei Chen, Ting Wang, An Li

**Affiliations:** 1 The Fourth Sub-Institute, Nuclear Power Institute of China, Chengdu 610213, China; 18328308347@163.com (P.D.);; 2 School of Materials Science and Engineering, Harbin Institute of Technology, Harbin 150001, China; liwanyinghit@163.com (W.L.);; 3 National Key Laboratory for Precision Hot Processing of Metals, Harbin Institute of Technology, Harbin 150001, China; 4 School of Materials Science and Engineering, Northwestern Polytechnical University, Xi’an 710072, China

**Keywords:** UN, surface adsorption, first-principles, nitrogen desorption, carbon doping

## Abstract

This work employs first-principles calculations to investigate the adsorption of nitrogen atoms and nitrogen molecules on the uranium mononitride (UN) surface and the desorption behavior of nitrogen atoms, and to construct a surface nitrogen vacancy stability boundary diagram. The UN(001) surface is identified as the thermodynamically most stable low-index surface. Nitrogen atoms preferentially adsorb at hollow sites, while the most stable N_2_ adsorption configuration has one N atom at a hollow site and the other bonded to a neighboring U atom. Thermodynamic analysis shows that under high temperature and low nitrogen partial pressure, nitrogen atoms tend to desorb from the UN surface. The effect of carbon doping on the stability of the UN surface was further investigated. For the configurations considered, as the carbon content increases, the desorption energy of N atoms generally increases, and the surface nitrogen vacancy stability region on UN(001) is significantly enlarged, thereby effectively suppressing the desorption of N atoms.

## 1. Introduction

Nuclear energy, as a clean, efficient, and sustainable power source, is playing an increasingly important role in the global energy structure. The advancement of reactors depends critically on the performance of nuclear fuels. Uranium mononitride (UN) is considered a promising candidate material for next-generation accident-tolerant fuels and space reactor fuels due to its high heavy metal atom density, excellent thermal conductivity, and high melting point [1,2,3,4,5]. However, UN is prone to nitrogen loss and decomposition under high-temperature and low-nitrogen-partial-pressure conditions, and its oxidation resistance is poor, which severely limits its engineering applications [6,7,8,9]. Therefore, a deep understanding of the interaction between UN surfaces and nitrogen, as well as exploring ways to enhance its thermal stability through doping, is of great scientific significance and engineering value.

Experimental phase diagrams have established the dominant role of nitrogen partial pressure in UN stability. Benz et al. [10], Hoenig [11], and Bugl et al. [12] found that the UN single-phase region is extremely narrow, decomposing at high temperature under low nitrogen pressure and remaining stable only under high nitrogen pressure. However, macroscopic phase diagrams cannot reveal the microscopic mechanism of surface nitrogen loss. DFT studies provide important atomic-scale insights [13]. Bocharov et al. [14] investigated point defects on the UN(001) surface and sub-surface layers, finding that N vacancies form more easily on the surface and that the surface vacancy formation energy is lower than that in the bulk, indicating that the surface is the primary region for nitrogen loss. Chen et al. [15] showed by microkinetic analysis that N_2_ is the dominant adsorbate on the UN surface at room temperature, and that nitrogen molecules in air can protect UN from oxidation, highlighting the protective role of N_2_ adsorption on surface stability. Bocharov et al. [16] further examined the interaction of O with surface N vacancies, finding that O atoms easily incorporate into surface N vacancies and are highly mobile, leading to UN oxidation, which in turn indicates that the presence of N vacancies weakens surface stability. Together, these studies indicate that N vacancy formation and N_2_ adsorption are key to understanding UN surface behavior. However, the thermodynamic correlation between lattice N atom desorption and temperature and nitrogen partial pressure has not been systematically established, and a quantitative link from microscopic desorption energy to macroscopic phase stability is still lacking.

During the preparation and service of UN, carbon is inevitably introduced as a common impurity element. Experimental studies have shown that UN and UC can form a complete solid solution, UC_1−x_N_x_ [17]. Besmann et al. [18] reviewed the phase equilibria and thermochemical data of the U–C–N system, successfully constructed isothermal section phase diagrams of the U–C–N system, and calculated the nitrogen partial pressures in the relevant regions. Ikeda et al. [19] and Prins et al. [20] measured the N_2_ decomposition pressure of UC_1−x_N_x_ and found that it decreases with decreasing x, accompanied by a decrease in the activity of UN in UC_1−x_N_x_. The preparation of U(N,C) solid solution is considerably easier than that of any single constituent phase [19]. Denise Adorno Lopes et al. [21] found through first-principles calculations that C atoms energetically prefer to occupy N vacancies in the UN lattice, and their diffusion behavior exhibits strong flux-coupling anisotropy. However, there remains a lack of systematic and in-depth studies on how carbon specifically affects the adsorption and desorption behavior of N_2_ on UN surfaces/in the bulk and the critical conditions.

In view of the above research progress, this work employs first-principles calculations to study the adsorption behavior of N atoms and N_2_ molecules as well as the desorption behavior of N atoms on the UN(001) surface. A surface nitrogen vacancy stability boundary diagram was constructed using thermodynamic criteria, and the quantitative relationship between critical nitrogen partial pressure and temperature was obtained by fitting with the Clausius–Clapeyron equation. On this basis, the effect of different carbon doping contents on the desorption behavior of N atoms was further investigated, and the critical nitrogen partial pressure–temperature relationships for various carbon contents were established. This provides a theoretical basis for the optimized design of uranium carbonitride fuels.

## 2. Materials and Methods

All first-principles calculations in this work were performed using the Vienna Ab-initio Simulation Package (VASP) 6.3.2 [22] within the framework of the projector augmented wave (PAW) method. The exchange-correlation energy was treated using the Perdew–Burke–Ernzerhof (PBE) functional under the generalized gradient approximation (GGA) [23]. The GGA-PBE functional was employed without the Hubbard U correction. We compared the lattice constants, magnetic moments, and phonon spectra with and without U, and found that the results without U agree better with experiment; therefore, GGA-PBE was selected. A detailed discussion is provided in the Supplementary Information. The plane-wave cutoff energy was set to 550 eV, and the convergence criteria for total energy and atomic forces were 10^−6^ eV and 10^−2^ eV/Å, respectively. Brillouin-zone sampling was performed using the Monkhorst–Pack scheme with a k-point spacing of 0.02 Å^−1^. The U(N,C) solid solution models were constructed using the SQS method implemented in the ATAT software 3.36 [24]. First, the UN bulk material was expanded to a 2 × 2 × 3 supercell containing 96 atoms in total. Then, 1, 2, 4, 6, and 8 N atoms were randomly replaced by C atoms, and the corresponding C doping concentrations were calculated using Equation (1). The (001) surface was cleaved from the relaxed bulk model, with a 2 × 2 surface supercell. When cleaving the surface, the cleavage position was chosen so that the C atoms were preferentially located in the outermost surface layer. The SQS method aims to approximate the statistical average properties of a random solid solution within a finite supercell; however, it may also lose important physical information related to local configurational effects. All surface models in this work contain six atomic layers, with the bottom two layers fixed during calculations. For N atom adsorption, 2 × 2, $\sqrt{2}\times \sqrt{2}$, and 1 × 1 supercells were used for coverages of 0.125, 0.25, and 0.5 ML, respectively. For N_2_ adsorption, a $\sqrt{2}\times \sqrt{2}$ supercell was used. A 15 Å vacuum layer was used in all cases. For all spin-polarized calculations, the initial magnetic moments of U atoms were set to 1.5 μB, and the converged average magnetic moment of U atoms was 1.20 μB. For the UN surface model, the ferromagnetic (FM) structure is generally considered more stable than the antiferromagnetic (AFM) structure [25,26]; therefore, the GGA functional with the FM structure was adopted for the UN surface calculations.

The mass fraction of C atoms was calculated using Equation (1).

(1) $$W_{c}=\frac{N_{C}M_{C}}{\sum_{i}N_{i}M_{i}}$$

where $N_{i}$ is the number of atoms of species i, and $M_{i}$ is the atomic mass of species i; i includes U, N, and C.

The surface energy was calculated using Equation (2).

(2) $$\gamma =\frac{1}{2A}\left(E_{surf}-N_{U}\mu_{U}-N_{N}\mu_{N}\right)$$

where *A* is the lateral area of a single surface supercell. *E_surf_* is the total energy of the surface model. *N* is the number of atoms. *μ* is the chemical potential of an atom in bulk UN.

The adsorption energy was obtained from Equation (3).

(3) $$E_{ads}=E_{tot}-E_{surf}-E_{N/N_{2}}$$

where *E_tot_* is the total energy of the adsorbed system. $E_{N/N_{2}}$ is the energy of an isolated N atom or N_2_ molecule [27].

The desorption energy of N atoms was calculated using Equation (4).

(4) $$E_{des}=E_{after}-E_{before}+\frac{1}{2}E_{N_{2}}$$

where *E_after_* and *E_before_* are the total energy of the system after and before desorption, respectively.

The Gibbs free energy of desorption of a nitrogen atom from the UN surface was calculated using Equation (5).

(5) $$G_{des}\left(T,p\right)=E_{after}-E_{before}+\mu_{N}\left(T,p^{0}\right)+\frac{1}{2}kTln\left(\frac{p}{p^{0}}\right)$$

where $\mu_{N}\left(T,p^{0}\right)$ is the chemical potential of nitrogen at temperature *T* and reference pressure p^0^ = 1 atm. *k* is the Boltzmann constant.

The chemical potential of nitrogen was calculated using Equation (6).

(6) $$\mu_{N}\left(T,p^{0}\right)=\frac{1}{2}E_{N_{2}}+\frac{1}{2}\left[H\left(T,p^{0}\right)-H\left(0K,p^{0}\right)\right]-\frac{1}{2}T\left[S\left(T,p^{0}\right)-S\left(0K,p^{0}\right)\right]$$

where *H* is the enthalpy of N_2_, and *S* is the entropy of N_2_. The values of *H* and *S* were taken from the JANAF thermochemical tables at 1 atm [28]. The $\mu_{N}\left(T,p^{0}\right)$ at different temperatures, derived from Equation (5), is listed in Table 1. Zero-point energy is not included in this chemical potential.

## 3. Results

### 3.1. N Adsorption and Desorption Processes on UN Surfaces

The low-index surfaces of UN, namely the (001) and (110) surfaces, were modeled. The surface energies of the two crystal orientations were calculated and compared, as shown in Table 2. The surface energies calculated in this work are higher than those reported in other studies; however, consistent with the literature, the surface energy of UN(001) is lower than that of UN(111). Therefore, the UN(001) surface was adopted in the subsequent study of N adsorption and desorption processes.

**Figure 1:**
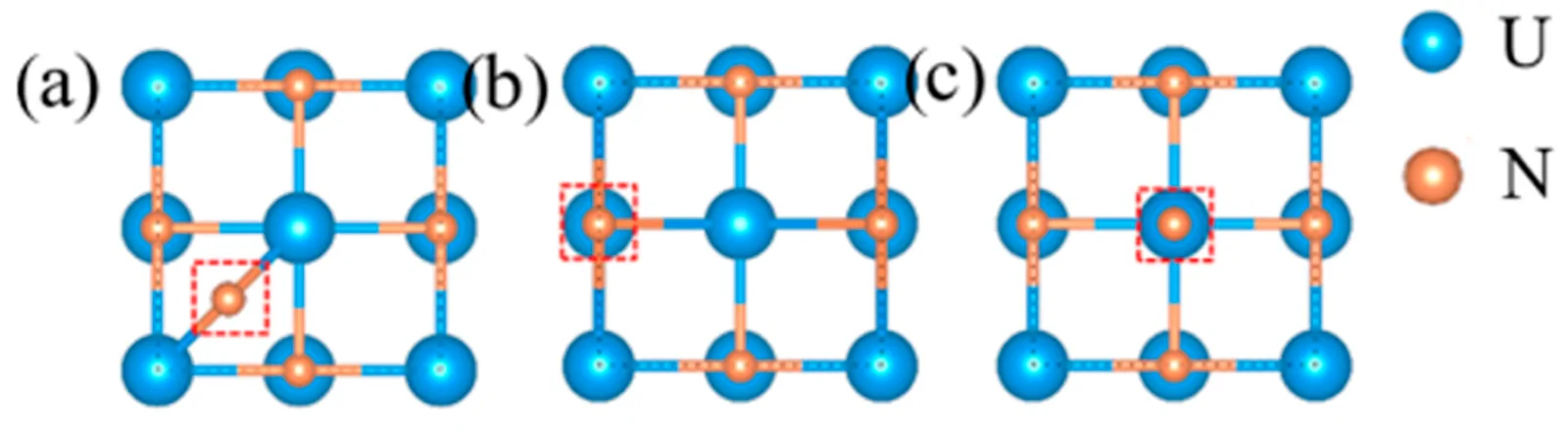
Schematic diagram of different adsorption sites for N atoms on the UN(001) surface. (**a**) Hollow site; (**b**) N-top site; (**c**) U-top site.

**Figure 2:**
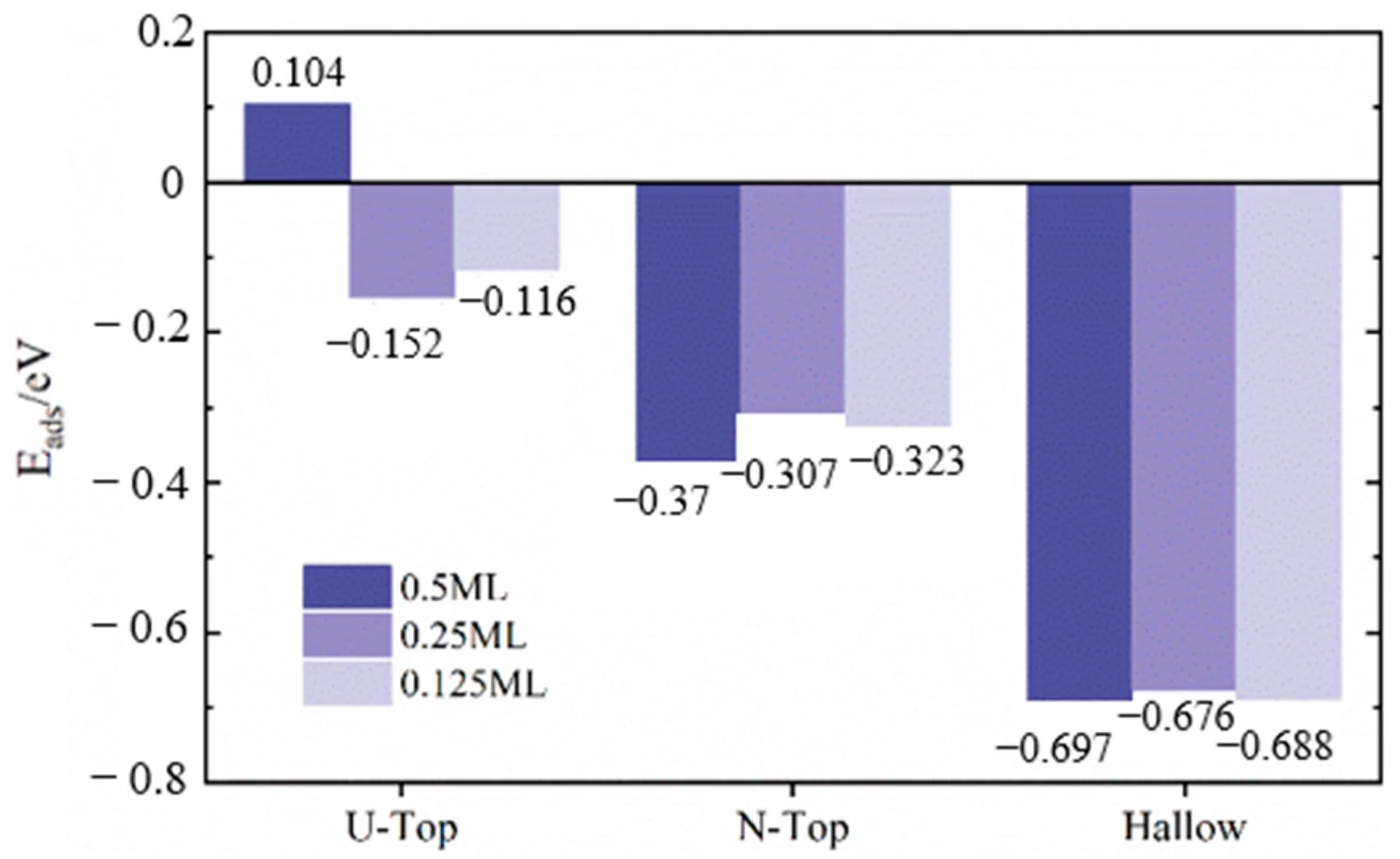
Effects of different surface coverages and adsorption sites on the adsorption energy.

The adsorption behavior of N atoms on the UN(001) surface was investigated. There are three different adsorption sites for N atoms on the UN(001) surface: the U-top site, the N-top site, and the four-fold Hollow site between U and N atoms, as shown in Figure 1. The adsorption energies of N atoms at different sites on the UN(001) surface under various coverages were calculated and are presented in Figure 2. The main difference among the three adsorption sites lies in the local environment around the N atom. The Hollow site is located in a cavity surrounded by four surface atoms, so it has more space and can bond with multiple neighboring U atoms. This helps saturate surface dangling bonds, making adsorption at this site the most stable. As coverage increases, its adsorption energy changes only slightly. The U-Top site is directly above a U atom and can bond with only one U atom, making it relatively crowded. At higher coverage, adjacent adsorbed N atoms get closer, leading to stronger repulsion, so the energy increases markedly and even becomes positive at high coverage, indicating that this site is highly unfavorable. The N-Top site is directly above a surface N atom, where N–N repulsion makes it less stable than the Hollow site. However, the surrounding U atoms can still provide some weak bonding, so its adsorption energy lies between those of the Hollow and U-Top sites.

**Figure 3:**
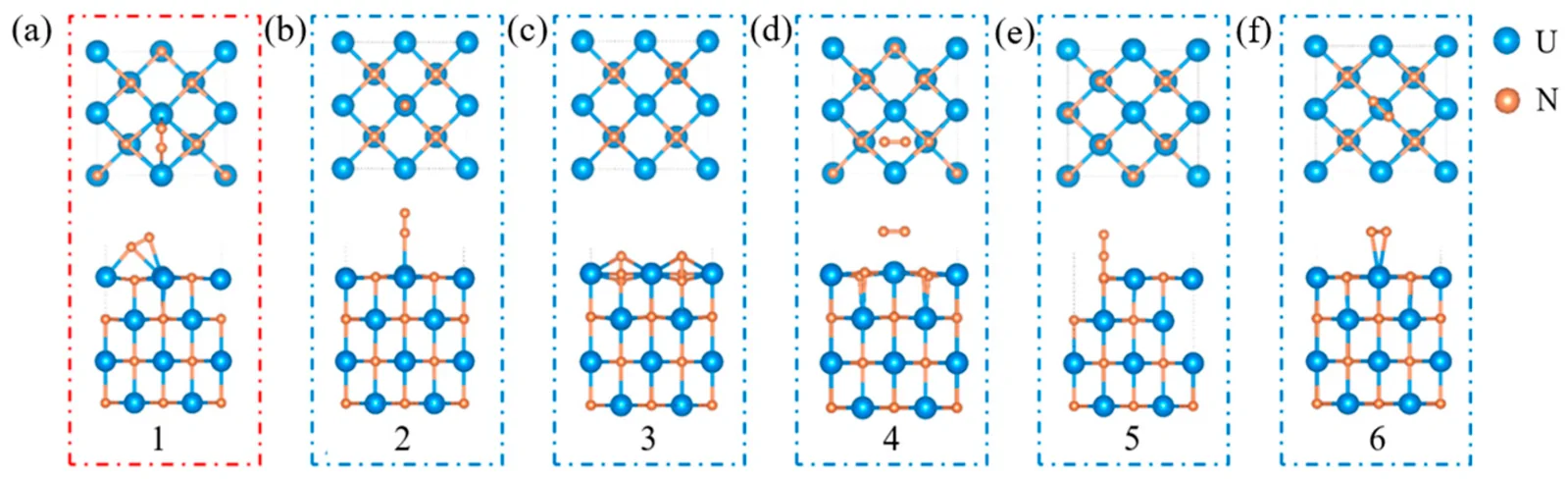
Different adsorption configurations of N_2_ on the UN(001) surface. (**a**) vertical adsorption at the Hollow site; (**b**) vertical adsorption at the U-top site; (**c**) horizontal adsorption at the N-top site; (**d**) horizontal adsorption at the Hollow site; (**e**) vertical adsorption at the N-top site; (**f**) horizontal adsorption at the U-top site.

**Figure 4:**
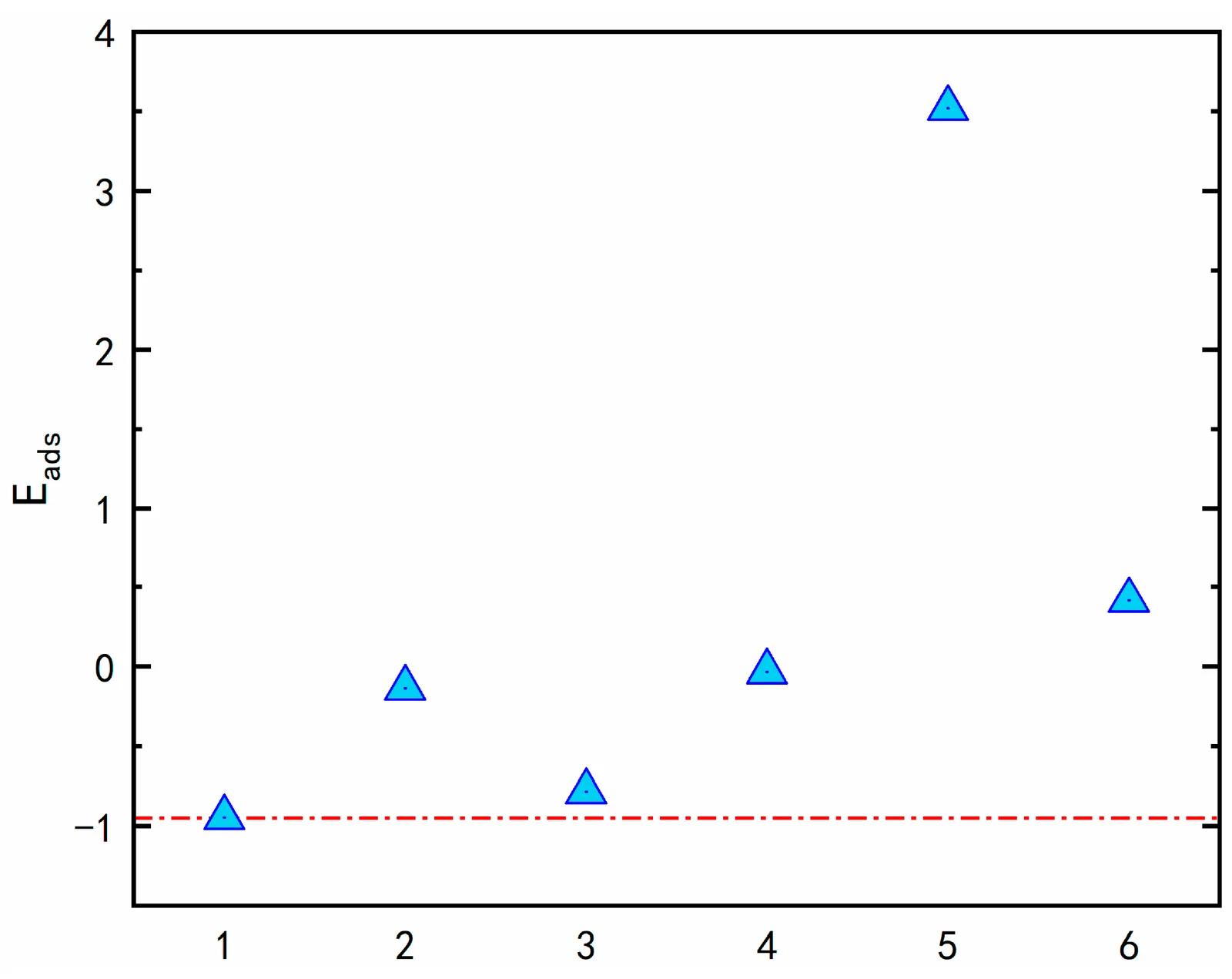
N_2_ adsorption energies of different adsorption configurations on the UN(001) surface.

The adsorption behavior of N_2_ molecules on the UN(001) surface was further investigated. As a diatomic molecule, the adsorption orientation of N_2_ can significantly influence its orbital coupling and charge transfer with the surface. Two extreme orientations of the molecular axis relative to the surface, namely horizontal and vertical adsorption, were considered, with the configuration schematics shown in Figure 3. The adsorption energies of N_2_ in various configurations were calculated by combining these orientations with different adsorption sites, and the results are presented in Figure 4. The adsorption energies for the vertical adsorption at the N-top site and the horizontal adsorption at the U-top site are positive, indicating that these processes are endothermic reactions. All other adsorption configurations exhibit exothermic characteristics. Configuration 1 is the most stable adsorption configuration, which is highlighted by a red box in Figure 3. In this configuration, both N atoms interact directly with the U surface: one N atom occupies the hollow site, while the other N atom binds to a neighboring U atom. The N≡N bond length is elongated to 1.65 Å. The N–U–N angle is approximately 30°. Meanwhile, the surface U–N bonds near the N_2_ adsorption site are significantly bent, indicating that N_2_ adsorption induces relaxation of the surface U–N framework. The neighboring U atoms shift toward N_2_, which further enhances the U–N_2_ interaction. Unlike the physisorption of N_2_ in other systems reported in previous studies [29,30], the adsorption of N_2_ on the UN(001) surface is significantly stronger and occurs via chemisorption through the formation of U–N bonds. This difference in binding mechanism leads to a much larger adsorption energy on the UN(001) surface and highlights the unique reactivity of the UN surface toward N_2_.

**Figure 5:**
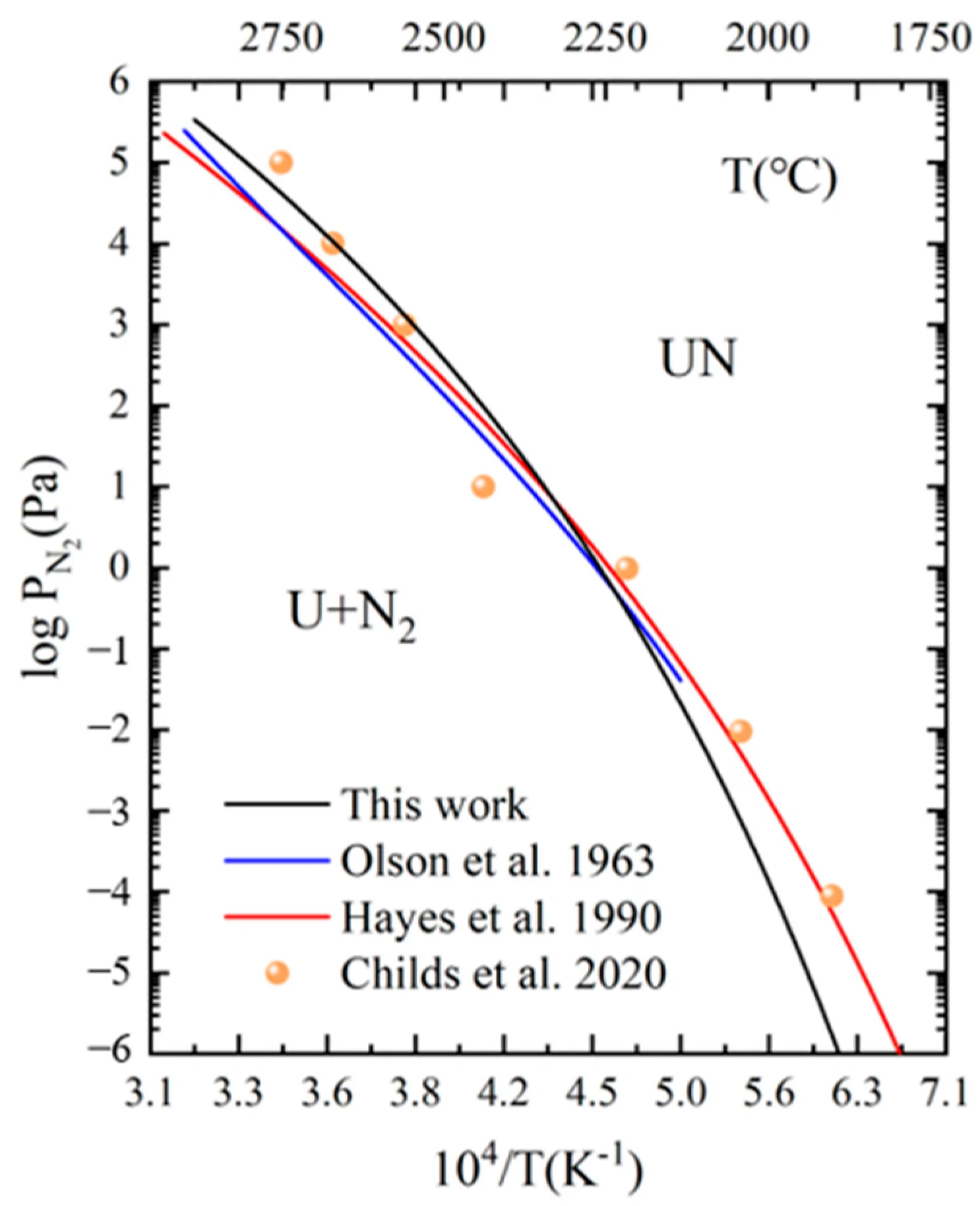
Stability diagram of N vacancies on the UN surface [35,36,37].

The desorption energies of N atoms from the UN(001) surface were calculated for different supercell sizes, as listed in Table 3. When the surface supercell was expanded to 2 × 2, the variation in desorption energy with surface size was already very small, with a difference of less than 0.01 eV, indicating that the interaction between N vacancies is negligible. Based on this converged desorption energy, the thermodynamic feasibility of N atom desorption from the UN(001) surface under various temperatures and nitrogen partial pressures was evaluated, and a surface nitrogen vacancy stability boundary diagram was constructed. The results were compared with the experimentally fitted curves reported in the literature, as shown in Figure 5. Under low nitrogen partial pressure, the effective binding of N atoms to the surface is weak, and the surface structure can exist stably only at sufficiently low temperatures. With increasing temperature, N atoms tend to escape from the surface, triggering the decomposition of UN. To suppress desorption, the nitrogen partial pressure must be correspondingly increased. Thus, the high-temperature stability of the UN surface is synergistically controlled by temperature and nitrogen partial pressure. Under conditions of high temperature and low nitrogen partial pressure, N atom desorption from the surface is thermodynamically feasible, causing the UN surface to become unstable and decompose; under conditions of low temperature and high nitrogen partial pressure, the UN surface remains stable and desorption is suppressed.

It should be emphasized that the quantity calculated in this work is the free energy of N atom desorption, and the boundary shown in Figure 5 is a surface nitrogen vacancy stability boundary, which does not automatically correspond to the experimentally measured phase boundary. However, the surface nitrogen vacancy stability boundary reflects the ease of phase decomposition. DFT calculations show that the formation energy of N vacancies on the UN(001) surface is significantly lower than that in the bulk, indicating that the surface is the region where nitrogen loss preferentially occurs [16]. Kinetic studies of UN high-temperature decomposition also indicate that the decomposition process begins with the release of gaseous nitrogen from the surface [31]. Moreover, differences in bulk thermal vacancy formation energies can drive atomic migration toward the surface, thereby inducing surface structural evolution and phase separation [32]. Therefore, the surface nitrogen vacancy stability boundary can be regarded as a microscopic indicator of the kinetically preferred initial step of overall phase decomposition. The vibrational free-energy contribution was not included in the present calculations, which may lead to a quantitative shift of the phase boundary at high temperatures [33,34]. This approximation does not affect the qualitative conclusions of this work regarding the trend of nitrogen vacancy formation and the effect of carbon doping; however, an accurate quantitative phase diagram would require further phonon calculations. Figure 5 presents the surface nitrogen vacancy stability boundary diagram and compares it with the experimental data from Refs. [35,36,37]. Above approximately 2000 K, the calculated boundary agrees well with the experimental data, indicating that the stability of nitrogen vacancies in the high-temperature region is mainly governed by nitrogen partial pressure. Below 2000 K, some deviation is observed between the calculated boundary and the curve fitted by Hayes et al. [36] based on experiments. Overall, the calculated results reasonably reproduce the basic experimental trend, demonstrating that the thermodynamic model employed in this work has good reliability in the high-temperature region. Finally, by fitting the surface nitrogen vacancy stability boundary curve in the figure according to the Clausius–Clapeyron equation, the quantitative relationship between the critical nitrogen partial pressure for the stable existence of the UN surface and temperature is obtained: ${log}_{10}P_{N_{2}}\left(Pa\right)=-\frac{38464.656}{T\left(K\right)}+9.337\times 10^{-4}lnT\left(K\right)+3.485\times 10^{-4}T\left(K\right)+16.85$.

**Table 3:** Desorption energies of N atoms from the UN(001) surface for different supercell sizes.

	1 × 1	$\sqrt{2}\times \sqrt{2}$	2 × 2
$E_{des}$ (eV)	3.483	3.631	3.621

### 3.2. N Atom Desorption Process on U(N,C) Surfaces

**Figure 6:**
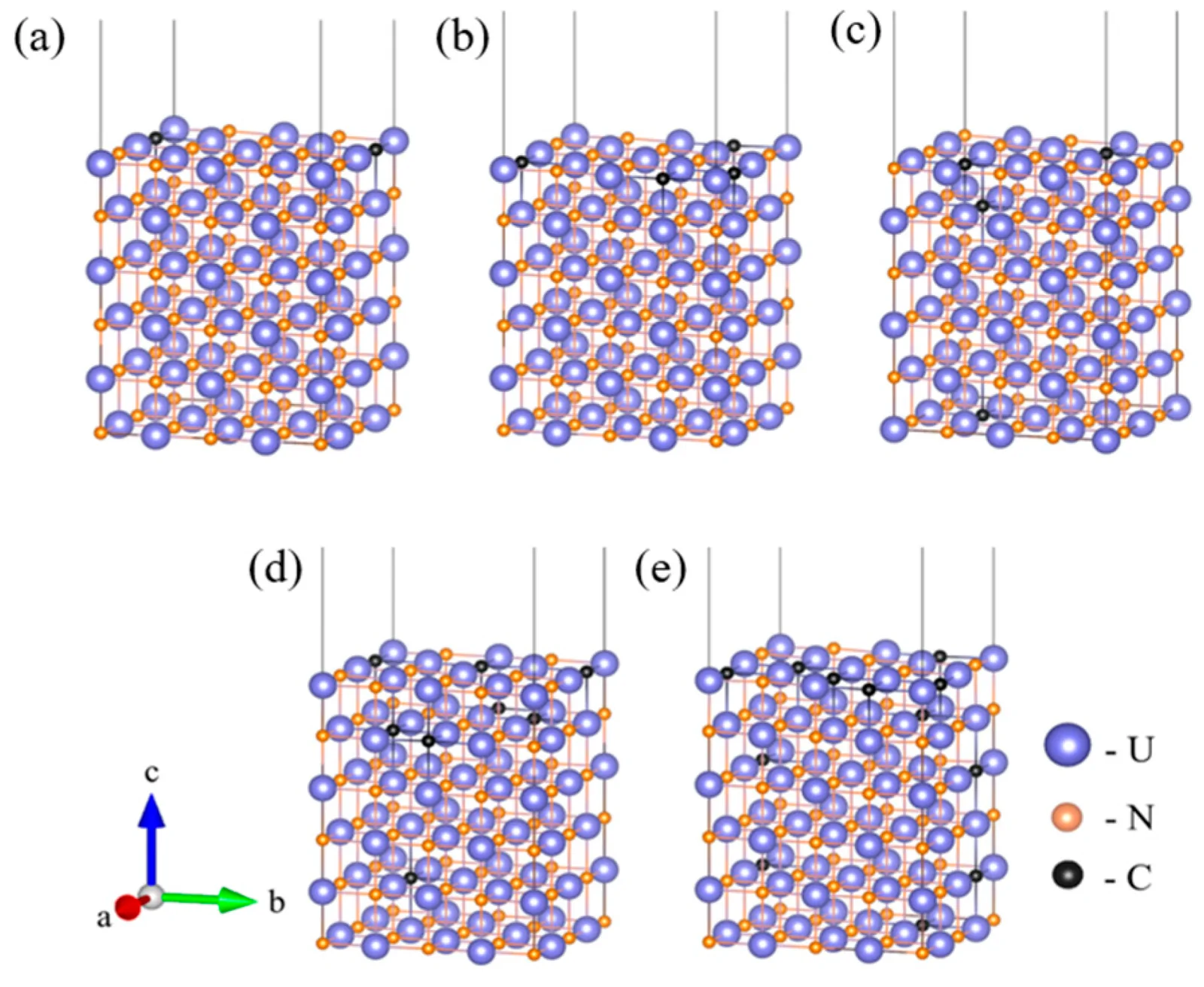
Schematic diagrams of the (001) surface models of the U(N,C) system with different C concentrations. (**a**) 0.1 wt% C; (**b**) 0.2 wt% C; (**c**) 0.4 wt% C; (**d**) 0.6 wt% C; (**e**) 0.8 wt% C.

**Figure 7:**
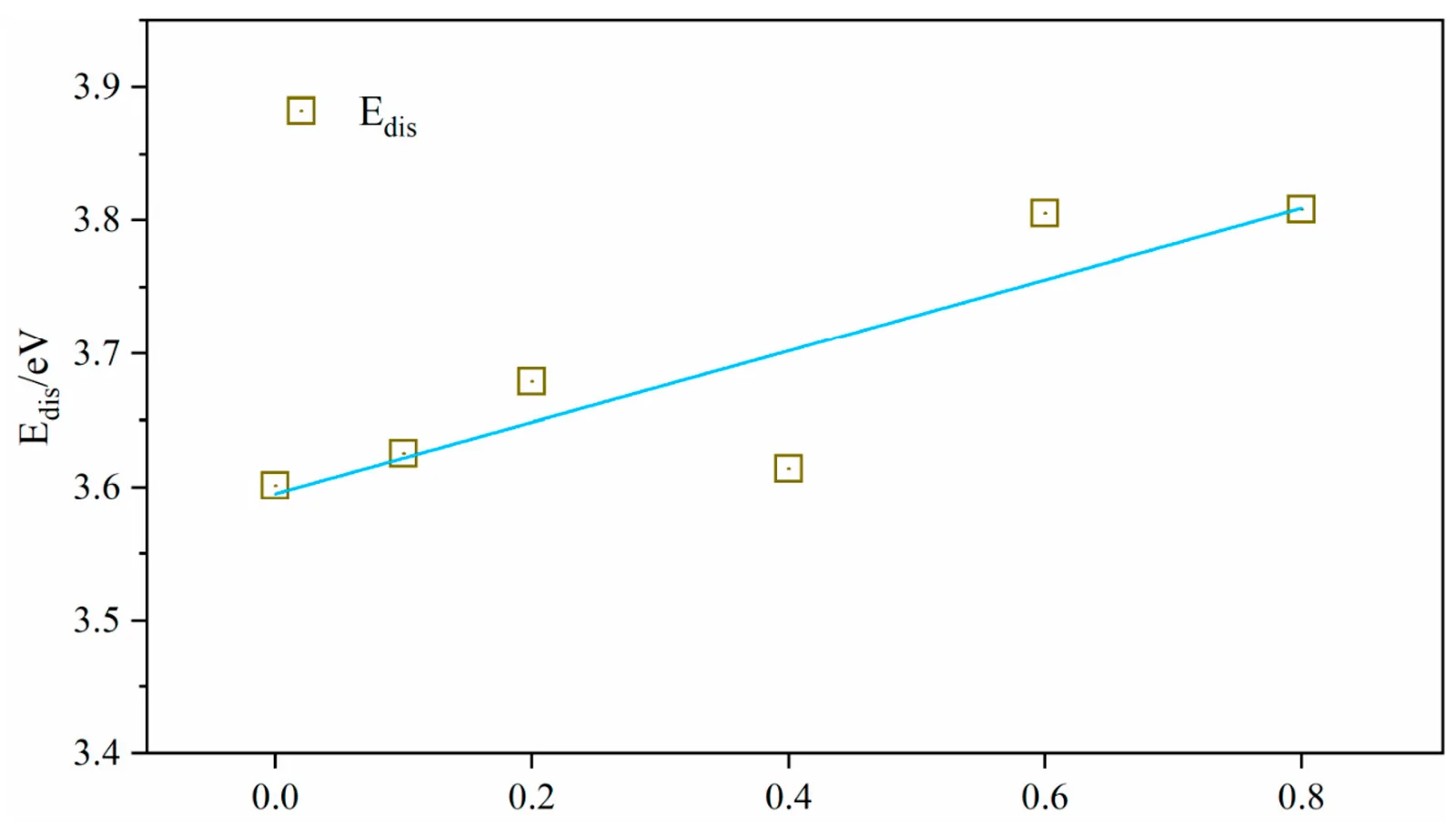
Desorption energies of N atoms in the (001) surface models of the U(N,C) system with different C concentrations.

Since the UN(001) surface exhibits the highest stability, we constructed five U(N,C)(001) surface models, as shown in Figure 6. It should be clarified that although carbon doping may change the relative stability of different facets, the present study mainly focuses on the (001) surface in order to compare with the properties of the undoped surface. The desorption energies of N atoms in the surface models with different carbon contents were calculated and are presented in Figure 7. It can be seen that as the carbon concentration increases, the desorption energy of N atoms also increases, implying that the decomposition of the UN surface becomes progressively more difficult at the same temperature.

**Figure 8:**
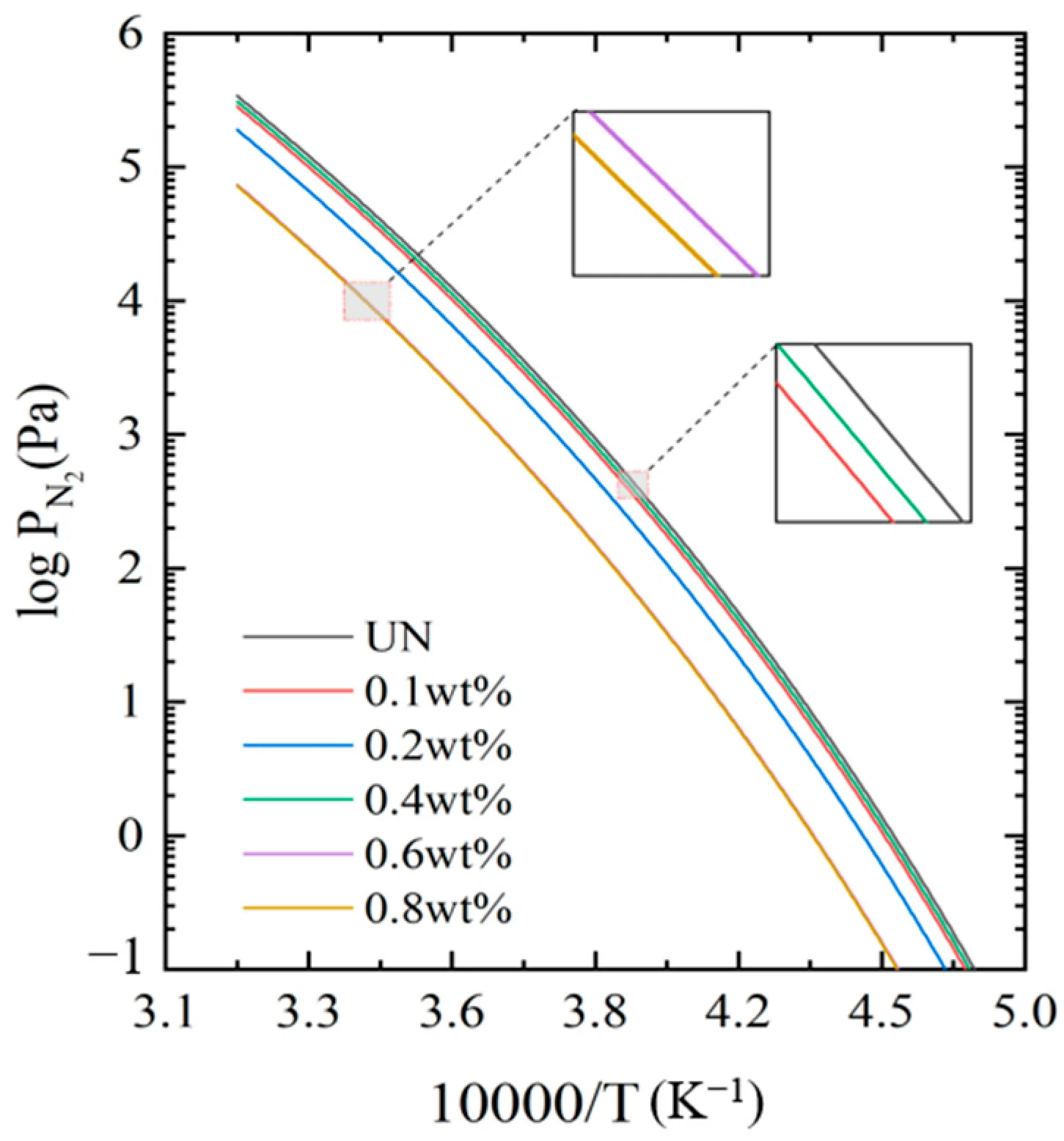
Temperature–nitrogen partial pressure phase diagram of U(N,C).

Based on the calculated desorption energies of nitrogen atoms in the U(N,C) systems with different carbon contents, the thermodynamic feasibility of N atom desorption from the (001) surface under various temperatures and nitrogen partial pressures was evaluated, and the surface nitrogen vacancy stability boundary diagrams for the U(N,C) systems were constructed, as shown in Figure 8. It can be seen that the introduction of carbon atoms shifts the boundary. Compared with pure UN, the surface nitrogen vacancy stability region of UN is enlarged for all carbon-containing systems, indicating that carbon doping can enhance the binding of nitrogen atoms on the surface, suppress their thermally induced desorption, and thereby expand the thermodynamically stable region of the UN surface nitrogen vacancy. This enhancement effect exhibits a clear dependence on carbon concentration: at low carbon contents (0.1 wt% and 0.4 wt%), the boundary shift is relatively limited, and the area of the surface nitrogen vacancy stability region changes little compared with that of pure UN; when the carbon content increases to 0.6 wt% and 0.8 wt%, the boundary shifts significantly toward lower nitrogen partial pressure and higher temperature, and the surface nitrogen vacancy stability region of UN is substantially broadened. The boundaries of each system were fitted according to the Clausius–Clapeyron equation, and the quantitative relationships between the critical nitrogen partial pressure and temperature at different carbon concentrations were obtained, as listed in Table 4. These relationships can be directly used to predict the maximum temperature for surface nitrogen vacancy stability under any given nitrogen partial pressure environment for a specific carbon content, providing a theoretical basis for the compositional design and operational control of uranium carbonitride fuels.

## 4. Conclusions

This work investigated the adsorption and desorption behavior of N on UN surfaces, as well as the effect of C content on the desorption of N from the UN surface. From a thermodynamic perspective, N atoms are found to preferentially adsorb at Hollow sites on the UN(001) surface. For N_2_ molecules, the configuration with one N atom located at a Hollow site and the other bonded to a neighboring U atom exhibits the lowest adsorption energy. The stability of the UN surface under high-temperature conditions is governed synergistically by temperature and nitrogen partial pressure. Under conditions of elevated temperature and low nitrogen partial pressure, the desorption of N atoms from the surface is thermodynamically favorable, leading to surface instability and decomposition. Carbon doping enhances the binding of nitrogen atoms on the surface and expands the thermodynamically stable region of the UN phase.

## Figures and Tables

**Table 1:** $\mu_{N}\left(T,p^{0}\right)$ over the temperature range 1500–3200 K at 1 atm.

*T* (K)	*μ_N_*(*T*, *p*^0^) (eV)	*T* (K)	*μ_N_*(*T*, *p*^0^) (eV)	*T* (K)	*μ_N_*(*T*, *p*^0^) (eV)
1500	−1.64	2100	−2.41	2700	−3.21
1600	−1.76	2200	−2.54	2800	−3.35
1700	−1.89	2300	−2.67	2900	−3.49
1800	−2.02	2400	−2.81	3000	−3.62
1900	−2.15	2500	−2.94	3100	−3.76
2000	−2.28	2600	−3.08	3200	−3.90

**Table 2:** Surface energies of different UN surfaces.

	(001)	(110)
γ (J/m^2^)	1.66, 1.458 [13], 1.37 [16]	2.470, 2.035 [13], 1.93 [16]

**Table 4:** Quantitative relationship between critical nitrogen partial pressure and temperature at different carbon concentrations.

Carbon Content (wt%)	Quantitative Relationship Between Critical Nitrogen Partial Pressure and Temperature
0.1	${log}_{10}P_{N_{2}}\left(Pa\right)=-\frac{38714.368}{T\left(K\right)}+1\times 10^{-3}lnT\left(K\right)+3.476\times 10^{-4}T\left(K\right)+16.854$
0.2	${log}_{10}P_{N_{2}}\left(Pa\right)=-\frac{39270.289}{T\left(K\right)}+8.608\times 10^{-4}lnT\left(K\right)+3.457\times 10^{-4}T\left(K\right)+16.867$
0.4	${log}_{10}P_{N_{2}}\left(Pa\right)=-\frac{38596.124}{T\left(K\right)}+1\times 10^{-3}lnT\left(K\right)+3.48\times 10^{-4}T\left(K\right)+16.852$
0.6	${log}_{10}P_{N_{2}}\left(Pa\right)=-\frac{40574.541}{T\left(K\right)}+1\times 10^{-3}lnT\left(K\right)+3.415\times 10^{-4}T\left(K\right)+16.888$
0.8	${log}_{10}P_{N_{2}}\left(Pa\right)=-\frac{40605.031}{T\left(K\right)}+8.48\times 10^{-4}lnT\left(K\right)+3.414\times 10^{-4}T\left(K\right)+16.891$

## Data Availability

The original contributions presented in this study are included in the article/Supplementary Materials. Further inquiries can be directed to the corresponding authors.

## Supplementary Materials

The following supporting information can be downloaded at: https://www.mdpi.com/article/10.3390/ma19204304/s1. Table S1. Equilibrium lattice constants (Å) and spin magnetic moments (μs) of U atoms in UN. Figure S1. Phonon spectrum of UN. References [38,39,40,41,42] are cited in Supplementary Materials.
