# Supplementary material for: A First-Principles Study on the Adsorption and Desorption of Nitrogen on UN and U(N,C) Surfaces
Source: Materials (Basel). 2026 Oct 13;19(20):4304. doi: 10.3390/ma19204304 (PMC13707218; doi:10.3390/ma19204304)
Supplement: Supplementary file 1 [file materials-19-04304-s001.zip › materials-4571705-supplementary.pdf]

The lattice parameters, U magnetic ordering, and average spin magnetic moments of U atoms were compared after structural optimization with GGA and GGA+U. Although the AFM phase predicted by GGA+U is consistent with experiment, the crystal structure of the AFM phase is expanded and distorted, and the predicted equilibrium stable structure exhibits a rectangular shape. In contrast, the FM phase given by PBE is cubic, and its lattice constant is closest to the experimental result (with an error of ~0.5%). The spin magnetic moment μ_S calculated by PBE agrees with experiment. GGA+U significantly overestimates the μ_S of the AFM phase, which is related to the distortion of the cubic structure in the AFM phase, indicating that GGA+U does not perform well for UN.

Table S1. Equilibrium lattice constants (Å) and spin magnetic moments (μ_s) of U atoms in UN.

		**a**	**b**	**c**	**μ_s**
GGA	FM	4.861	4.861	4.861	1.20
GGA+U	AFM	5.028	4.902	4.9682	1.89
Expt.[1]	AFM	4.889	-	-	0.75
GGA[2]	FM	4.861	4.861	4.861	1.21
GGA+U[2]	AFM	5.009	4.898	4.956	1.83

The phonon spectrum of the FM phase calculated using GGA is in good agreement with the experimental phonon spectrum, and also agrees well with the GGA phonon spectrum previously reported by Vancho et al.[3].

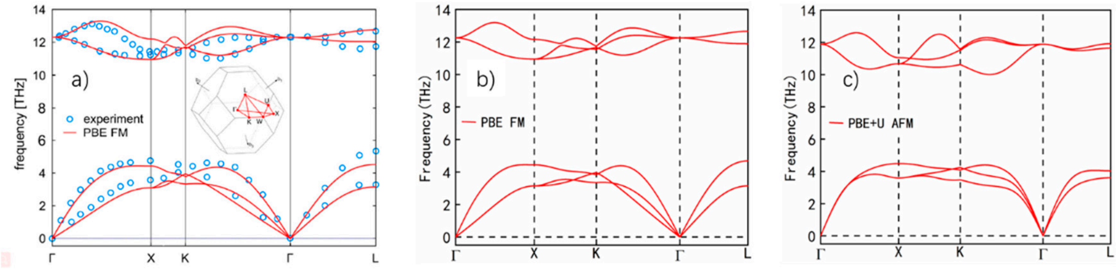

Figure S1. Phonon spectrum of UN.
(a) Phonon spectrum calculated by Vancho et al.[4] and experimental data from J. A. Jackman et al.[5] (circles); (b) phonon spectrum calculated with GGA; (c) phonon spectrum calculated with GGA+U.

[1] Staun Olsen J, Gerward L, Benedict U. A new high-pressure phase of uranium nitride studied by X-ray diffraction and synchrotron radiation[J]. Journal of Applied Crystallography, 1985, 18(1): 37-41.

[2] Kocevski V, Rehn D A, Cooper M W D, et al. First-principles investigation of uranium mononitride (UN): Effect of magnetic ordering, spin-orbit interactions and exchange correlation functional[J]. Journal of Nuclear Materials, 2022, 559: 153401.

[3] Jomard G, Amadon B, Bottin F, et al. Structural, thermodynamic, and electronic properties of plutonium oxides from first principles[J]. Physical Review B, 2008, 78(7): 075125.

[4] Mei Z G, Stan M, Pichler B. First-principles study of structural, elastic, electronic, vibrational and thermodynamic properties of UN[J]. Journal of Nuclear Materials, 2013, 440(1-3): 63-69.

[5] Jackman J A, Holden T M, Buyers W J L, et. al. Systematic study of the lattice dynamics of the uranium rocksalt-structure compounds[J]. Physical Review B, 1986, 33(10): 7144-7153.
